# Effects of nebivolol versus other antihypertensive drugs on the endothelial dysfunction in patients with essential hypertension

**DOI:** 10.1042/BSR20200436

**Published:** 2020-05-05

**Authors:** Bingwei Li, Qiuju Zhang, Honggang Zhang, Chunxiao Wang, Ruijuan Xiu

**Affiliations:** Institute of Microcirculation, Chinese Academy of Medical Sciences (CAMS) & Peking Union Medical College (PUMC), Beijing 100005, China

**Keywords:** β-blockers, endothelial dysfunction, hypertension, Nebivolol

## Abstract

We aim to determine whether nebivolol has a better effect on endothelial dysfunction compared with other β-blockers or other classes of antihypertensive drugs. Searches of the PubMed, Embase etc. were performed to analyze all the randomized controlled trials using nebivolol to treat essential hypertension. The primary end points included a measurement of peripheral endothelial function by brachial flow mediated vasodilatation (FMD) or forearm blood flow (FBF). A random-effect model was used to perform the meta-analysis when the studies showed significant heterogeneity, otherwise a descriptive analysis was conducted. Ten studies (689 patients) were included in qualitative analysis, four of which were included in quantitative synthesis. Meta-analysis showed that the changed FMD value before and after treatment with nebivolol was not statistically different from those treated with other β-blockers [mean difference = 1.12, 95% confidence interval (CI): −0.56, 2.81, *P*=0.19]. Descriptive analysis indicated that nebivolol did not have a better endothelium-protective effect than other classes of antihypertensive drugs including olmesartan and perindopril. Nebivolol is not a unique endothelial function-protective agent distinguished from other β-blockers or other classes of antihypertensive drugs. Reversal of endothelial dysfunction is a key point in the prevention and therapy of essential hypertension.

## Introduction

Endothelial dysfunction is increasingly observed in human essential hypertension, which is a common risk factor for other cardiovascular diseases including strokes, heart attacks, and atherosclerosis [1–3]. Endothelial dysfunction is used to describe the impairment of its vasodilatory capacity, due to the changes in mechanical and biological barrier established by endothelial cells and the imbalance of endothelium-derived relaxing and constricting factors [4,5]. Although the explicit cause–effect relationship between endothelial dysfunction and essential hypertension needs to be elucidated [6], endothelial dysfunction is an independent predictor for the future cardiovascular events in the patients with hypertension, and patients with improved endothelial function have a better prognosis compared with those possessing impaired endothelial function [7,8]. Therefore, therapy focused on rectifying endothelial dysfunction may be beneficial for the patients with essential hypertension.

Brachial flow-mediated vasodilatation (FMD) is the commonest, low-risk, and non-invasive method to evaluate the endothelial dysfunction [9]. FMD is mainly mediated by endothelium-derived nitric oxide (NO) when the brachial artery responds to an increased blood flow-induced change of shear stress [10]. FMD value is commonly calculated as a percentage of change in the diameter of the brachial artery using ultrasonography, in which reactive hyperemia is reached via inflation and deflation of a sphygmomanometer cuff [11]. Changed FMD, a percentage of change between after and before treatment, could be used to quantitatively reflect the effect of a medicine on the endothelial function of the patients. A meta-analysis with moderate methodological quality involving 5547 participants indicates that impaired brachial FMD is significantly relevant to the development of cardiovascular outcomes [12]. Forearm blood flow (FBF) is an invasive measurement to evaluate the endothelial dysfunction by venous occlusion plethysmography (VOP) [13]. Similarly, the dilatation of the peripheral arteries in FBF method was also induced by reactive hyperemia with a blood pressure cuff, and the change in the FBF values were recorded [14]. This invasive method is more commonly used before the development and regular use of FMD [15].

Multiple antihypertensives including some β-blockers have been reported to alleviate endothelial dysfunction except for their blood pressure-lowering effect [9]. Nebivolol is not only a β1-adrenoceptor blocker, but a β3-adrenoceptor agonist exerting multiple effects including vasodilation and antioxidation [16]. This characteristic of nebivolol may provide a particular endothelium-protective effect. Nebivolol may have better effects on endothelial dysfunction due to enhanced NO bioavailability compared with other β-blockers [17,18].

We designed the current study to determine whether nebivolol has a better effect on endothelial dysfunction compared with other β-blockers or other classes of antihypertensive drugs.

## Materials and methods

### Search strategy

We electronically searched the medical databases including PubMed, Embase, Web of Science, Cochrane Central Register of Controlled Trials (CENTRAL), Clinical Trials, and WHO International Clinical Trials Registry Platform (ICTRP) (through 30 April 2018). In addition, we also searched the reference lists of retrieved studies and manual search was supplemented as well to identify articles missed in primary searches. The search strategy was shown in Table 1.

**Table 1:** Research strategy of the present study

Number	Search strategy
#1	High blood pressure OR hypertension OR ‘Hypertension’[Mesh]
#2	Nebivolol OR nebirol OR Bystolic OR Lobivon OR ‘Nebivolol’[Mesh] OR ME-3255 OR R-65824 OR R-67555 OR C07AB12 OR Nebilet
#3	Randomized OR randomised OR randomization OR randomisation OR randomly OR random OR ‘Random Allocation’[Mesh] OR ‘Randomized Controlled Trial’ [pt]
#4	#1 AND #2 AND #3

### Study selection

Studies included in this meta-analysis (through 30 April 2018) had similar methodology and comprehensive outcome measures. Included trials met the following inclusion criteria: (1) Randomized controlled trials (RCTs) or randomized crossover trials. (2) Patients with essential hypertension diagnosed with the standard definition and clinical guideline of hypertension. (3) Trials compared nebivolol with no treatment or placebo, or compared nebivolol with other β-blockers or other antihypertensives. (4) The primary end points included a non-invasive measurement of peripheral endothelial function by FMD or an invasive measurement by FBF.

### Risk of bias

Cochrane Collaboration’s tool was used to evaluate the risk of bias for all the articles [19]. A total of six domains of bias including selection bias, performance bias, detection bias, attrition bias, reporting bias, and other bias were evaluated. The biases are categorized in the format of low, high, or unclear risk.

### Data extraction

The articles identified in above databases were screened and the duplicates were discarded by a reference management software. Two investigators independently filtered and evaluated the literature according to the inclusion criteria. Using the PICOS principle, one investigator extracted the data including basic characteristics, study types, subjects of the study, risk factors and outcomes based on the predesigned table. The other investigator independently reviewed and checked the data. Disagreements were resolved by discussions or consultation with a third-party statistician.

### Statistical analysis

RevMan 5.3 software, which was provided by the Cochrane Collaboration, was used to synthesize the results and perform the meta-analysis. Continuous variables are presented as mean difference and 95% confidence interval (CI). Chi square (*χ^2^*) test was performed to evaluate the heterogeneity between studies, and *P*≤0.1 was considered as statistically significant. The varied heterogeneity between studies was defined according to the value of *I^2^* as high (≥75%), moderate (≥50%, <75%), and low (≥25%, <50%). A random-effect model was used to perform the meta-analysis when the studies showed significant heterogeneity. Descriptive analysis was conducted when the heterogeneity was too large or the data source was uncertain. The result of meta-analysis was shown in data and forest plot, respectively. A *P*-value <0.05 was considered statistically significant.

## Results

### Literature search

**Figure 1:**
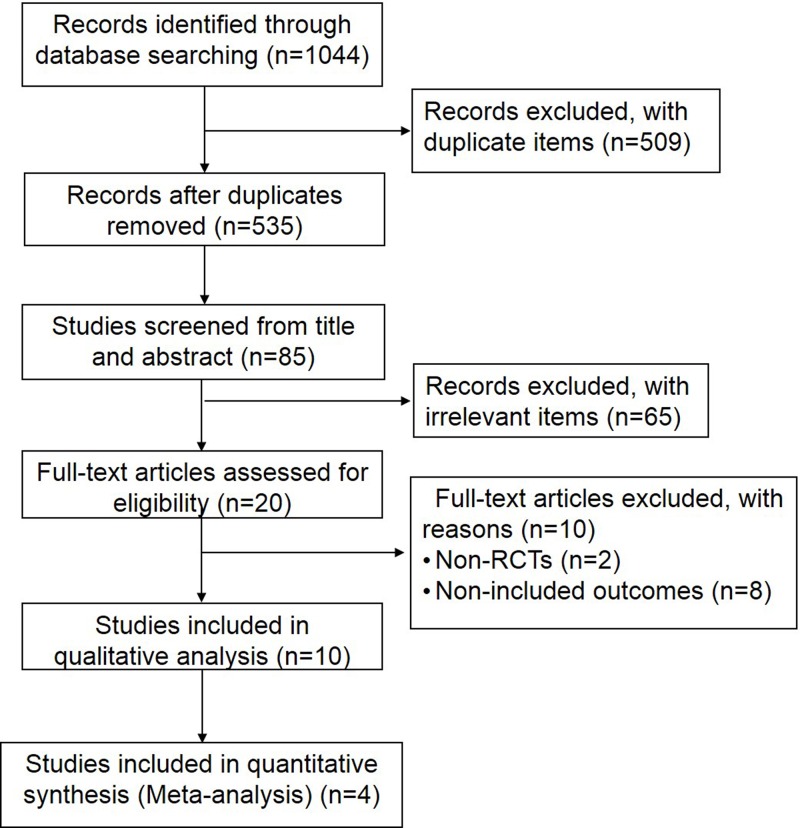
Flow diagram of search and selection

A total of 1044 potentially eligible studies were identified through database searching. After exclusion of the records with duplicate items, the studies (*n*=535) were further screened for the title and abstract, of which 450 studies were excluded. Then 20 full-text articles of the remaining 85 studies were assessed for eligibility after exclusion of irrelevant items. Finally, ten studies that met our inclusion criteria were included in qualitative analysis, four of which were included in quantitative synthesis (meta-analysis, Figure 1).

### Risk of bias

The risk of bias for each study was evaluated by Cochrane Collaboration’s tool. The main source of bias in current studies included unclear risk for random sequence generation, allocation concealment, and blinding of outcome assessment. A high risk for blinding of participants and researchers was also found in some included studies. The details of the bias evaluation were shown in Table 2.

**Table 2:** Risk of bias assessment

Author/year	Random sequence generation	Allocation concealment	Blinding of participants and researchers	Blinding of outcome assessment	Incomplete outcome data	Selective reporting
Neuman et al., 2016 [13]	Unclear	Unclear	Low	Unclear	Low	Low
Sendur et al., 2014 [11]	Unclear	Unclear	High	Unclear	Low	Low
Zepeda et al., 2012 [20]	Unclear	Unclear	High	Unclear	Low	Low
Espinola-Klein et al., 2011 [21]	Unclear	Unclear	Low	Unclear	Low	Low
Fedorishina et al., 2010 [22]	Unclear	Unclear	Unclear	Unclear	Low	Low
Pronko et al., 2009 [14]	Low	Unclear	High	Unclear	Low	Low
Simova et al., 2009 [26]	Unclear	Unclear	Low	Unclear	Low	Low
Pasini et al., 2008 [23]	Unclear	Unclear	Low	Unclear	Low	Low
Ghiadoni et al., 2003 [24]	Unclear	Unclear	High	Unclear	Low	Low
Tzemos et al., 2001 [25]	Unclear	Unclear	Low	Unclear	Low	Low

### Characteristics of included trials

Ten clinical trials and 689 patients were included in this analysis, of which 128 patients were randomized in one double-blind, parallel-group design trial, 90 randomized in three double-blind crossover design trials, 214 randomized in two single-blind, parallel-group design trials, 187 randomized in three-open design trials, and 70 randomized in one unreported design trial (Table 3). The patients received nebivolol, ranging from 5 mg [20] to 10 mg [13], or control antihypertensives including β-blockers (metoprolol [13,21], carvedilol [20,22], atenolol [14,23–25], bisoprolol [26]) or other antihypertensives (olmesartan [11], nifedipine [24], amlodipine [24], telmisartan [24], perindopril [24]). One of these trials compared the effects of combined use of nebivolol and enalapril with the combination of atenolol and enalapril [14], and another showed an effect difference between nebivolol and atenolol on the background of 2.5 mg bendrofluazide [25]. The treatment time ranged from 2 [14] to 48 [24] weeks. In two trials [13,25], FBF measurement, other than FMD, was obtained using a venous occlusion strain gauge plethysmograph to evaluate the endothelial function. The characteristics of the included trials are shown in Table 3.

**Table 3:** Characteristics of the included trials

Author/year	Country	Design	Masking	Treatment time	Nebivolol (*n*)	Nebivolol dose	Control antihypertensives	Control (*n*)	Control dose
Neuman et al., 2016 [13]	U.S.A.	Crossover	Double	12 w	19	10 mg	Metoprolol	19	100 mg
Sendur et al., 2014 [11]	Turkey	Parallel	Open	8 w	43		Olmesartan	42	
Zepeda et al., 2012 [20]	Chile	Parallel	Single	12 w	21	5 mg	Carvedilol	23	12.5 mg
Espinola-Klein et al., 2011 [21]	Germany	Parallel	Double	48 w	65	5 mg	Metoprolol	63	95 mg
Fedorishina et al., 2010 [22]	Russia	Parallel	-	8 w	25	5 mg	Carvedilol	45	25 mg
Pronko et al., 2009 [14]	Belarus	Parallel	Open	2 w	23	5 mg+20–40 mg enalapril	Atenolol	29	50 mg+20–40 mg enalapril
Simova et al., 2009 [26]	Bulgaria	Crossover	Open	8 w	25	5 mg	Bisoprolol	25	5 mg
Pasini et al., 2008 [23]	Italy	Crossover	Double	4 w	20	5 mg	Atenolol	20	100 mg
Ghiadoni et al., 2003 [24]	Italy	Parallel	Single	48 w	28	5–10 mg	Nifedipine GITS	28	30–60 mg
							Amlodipine	28	5–10 mg
							Atenolol	29	50–100 mg
							Telmisartan	29	80–160 mg
							Perindopril	28	2–4 mg
Tzemos et al., 2001 [25]	U.K.	Crossover	Double	8 w	6	5 mg+2.5 mg bendrofluazide	Atenolol	6	50 mg+2.5 mg bendrofluazide

In all the included trials, basic characteristics of the patients were comparable before treatments between groups. The age of the patients ranged from 30.0 [22] to 66.7 [21] years.

The mean systolic blood pressure (SBP) and mean diastolic blood pressure (DBP) ranged between ‘1 grade’ [22] ∼156 mmHg [24] and ‘1 grade’ [22] ∼102 mmHg [24] before treatments, respectively. In addition, the SBP and DBP were not statistically different after antihypertensive treatments between groups. The incidence of hypertensive risk factors including diabetes mellitus, dyslipidemia, and smoking were also comparable between treatment groups (Table 4).

**Table 4:** Characteristics of the patients and the distribution of risk factors

Author/year	Treatment	Age (y)	Male, *n* (%)	SBP (mmHg, before treatment)	SBP (mmHg, after treatment)	DBP (mmHg, before treatment)	DBP (mmHg, after treatment)	Diabetes mellitus, *n* (%)	Dyslipidemia, *n* (%)	Current smokers, *n* (%)	Former smokers, *n* (%)
Neuman et al., 2016 [13]	Nebivolol	51 ± 8.6	13 (68.4)		135 ± 15		81 ± 14	1 (5.3)	6 (31.5)	7 (36.8)	
	Metoprolol	51 ± 8.6	13 (68.4)		134 ± 15		81 ± 21	1 (5.3)	6 (31.5)	7 (36.8)	
Sendur et al., 2014 [11]	Nebivolol	50.1 ± 9.4	11 (25.6)	151.2 ± 4.1	132.3 ± 9.6	93.9 ± 2.5	84.8 ± 6.4			11 (25.6)	4 (9.3)
	Olmesartan	54.9 ± 7.9	19 (45.2)	154.2 ± 4.0	131.4 ± 11.8	94.9 ± 2.4	83.2 ± 2.4			11 (26.2)	9 (21.4)
Zepeda et al., 2012 [20]	Nebivolol	44.9 ± 2.1	15 (71.4)	141 ± 6.3	−17.4^1^	98.7 ± 5.2	−13.7^1^				
	Carvedilol	45.6 ± 2.8	16 (69.6)	139 ± 5.1	−19.9^1^	97.3 ± 6.6	−12.8^1^				
Espinola-Klein et al., 2011 [21]	Nebivolol	66.7 ± 8.3	45 (86.5)	147.6 ± 6.6	−5.2^1^	79.6 ± 7.4	−1.7^1^	17 (32.7)	31 (59.6)	15 (28.8)	33 (63.5)
	Metoprolol	65.9 ± 7.9	41 (71.9)	147.6 ± 6.6	−3.9^1^	81.4 ± 7.6	−2.5^1^	12 (21.1)	39 (68.4)	19 (33.3)	29 (50.9)
Fedorishina et al., 2010 [22]	Nebivolol	30–55		1–2 grades		1–2 grades					
	Carvedilol	30–55		1–2 grades		1–2 grades					
Pronko et al., 2009 [14]	Nebivolol		10 (43.5)	2–3 grades		2–3 grades		0 (0)	0 (0)	0 (0)	
	Atenolol		14 (48.3)	2–3 grades		2–3 grades		0 (0)	0 (0)	0 (0)	
Simova et al., 2009 [26]	Nebivolol	45.3 ± 11.5	18 (72.0)	152.4 ± 18.5	131.8 ± 11.5	99.3 ± 9.3	82.4 ± 7.1	0 (0)	7 (28.0)	7 (28.0)	4 (16.0)
	Bisoprolol	45.3 ± 11.5	18 (72.0)	152.4 ± 18.5	129.7 ± 10.2	99.3 ± 9.3	83.1 ± 7.0	0 (0)	7 (28.0)	7 (28.0)	4 (16.0)
Pasini et al., 2008 [23]	Nebivolol	55.9 ± 10.0	8 (40.0)	152.4 ± 8.1	133.0 ± 7.2	96.1 ± 4.3	85.0 ± 3.1				
	Atenolol	55.9 ± 10.0	8 (40.0)	151.8 ± 7.7	134.2 ± 5.1	96.5 ± 5.1	85.8 ± 3.6				
Ghiadoni et al., 2003 [24]	Nebivolol	53 ± 8	17 (60.7)	152 ± 9	136 ± 10	98 ± 9	84 ± 6	0 (0)			
	Nifedipine GITS	52 ± 11	17 (60.7)	153 ± 8	137 ± 11	102 ± 2	87 ± 5	0 (0)			
	Amlodipine	53 ± 8	17 (60.7)	152 ± 9	136 ± 10	98 ± 9	84 ± 6	0 (0)			
	Atenolol	53 ± 9	18 (62.1)	156 ± 10	136 ± 10	99 ± 8	84 ± 6	0 (0)			
	Telmisartan	50 ± 9	18 (62.1)	151 ± 10	133 ± 10	100 ± 7	86 ± 5	0 (0)			
	Perindopril	51 ± 11	18 (64.3)	153 ± 9	134 ± 10	100 ± 6	86 ± 6	0 (0)			
Tzemos et al., 2001 [25]	Nebivolol	52 ± 7		154 ± 8	132 ± 7	98 ± 9	82 ± 6	0 (0)	0 (0)	0 (0)	
	Atenolol	52 ± 7		154 ± 8	132 ± 9	98 ± 9	83 ± 4	0 (0)	0 (0)	0 (0)	

1 Absolute change in blood pressure.

### Evaluation of endothelial function

Brachial FMD or FBF was used to evaluate the endothelial function of the hypertensive patients in the included ten clinical trials. Among them, non-invasive measurement (FMD) was used in seven trials [11,20–24,26] and invasive measurement (FBF) was used in three trials [13,14,25].

The values of brachial FMD ranged from 4.14 ± 3.55% [26] to 6.6 ± 3.1% [21] before treatment, and from 5.6 ± 2.4% [24] to 8.99 ± 4.21% [26] after treatment with nebivolol. In the groups of other control antihypertensives, these FMD values ranged from 4.14 ± 3.55% [26] to 6.8 ± 3.5% [21] before treatment, and from 3.72 ± 6.84% [26] to 8.0 ± 2.5% [11] after treatment. There was no original FMD value before and after treatment in two trials, in which only changed FMD value was reported (nebivolol vs carvedilol: 7.3 vs 8.1% and 1.6 vs 5.5%, respectively, Table 5).

**Table 5:** Measurements of endothelial function in the patients with essential hypertension

Author/year	FMD (%, before treatment)	FMD (%, after treatment)	Changed FMD (%)	Control antihypertensives	FMD (%, before treatment)	FMD (%, after treatment)	Changed FMD (%)	Notes
Sendur et al., 2014 [11]	5.9 ± 2.1	8.1 ± 2.7		Olmesartan	5.5 ± 2.1	8.0 ± 2.5		
Zepeda et al., 2012 [20]			7.3	Carvedilol			8.1	No SD
Espinola-Klein et al., 2011 [21]	6.6 ± 3.1	6.5 ± 3.3	−0.21	Metoprolol	6.8 ± 3.5	7.3 ± 3.8	0.52	No SD; SD was calculated by 95% CI
Fedorishina et al., 2010 [22]			1.6	Carvedilol			5.5	Only abstract; no SD
Simova et al., 2009 [26]	4.14 ± 3.55	8.99 ± 4.21		Bisoprolol	4.14 ± 3.55	3.72 ± 6.84		
Fratta Pasini et al., 2008 [23]	5.93 ± 1.9	7.52 ± 2.2		Atenolol	5.85 ± 2.1	6.11 ± 2.3		
Ghiadoni et al., 2003 [24]	5.3 ± 2.2	5.6 ± 2.4	0.5 ± 2.2	Nifedipine GITS	5.2 ± 2.1	4.8 ± 1.9	−0.5 ± 2.4	
				Amlodipine	5.4 ± 2.0	5.1 ± 1.8	−0.3 ± 2.5	
				Atenolol	5.5 ± 2.1	5.7 ± 1.9	0.4 ± 2.1	
				Telmisartan	5.5 ± 2.1	5.6 ± 1.9	0.3 ± 2.9	
				Perindopril	5.1 ± 2.0	6.4 ± 2.4	1.5 ± 2.1	

	FBF (%, before treatment)	FBF (%, after treatment)	Changed FBF (%)	Control antihypertensives	FBF (%, before treatment)	FBF (%, after treatment)	Changed FBF (%)	
Neuman et al., 2016 [13]			21	Metoprolol			12	
Pronko et al., 2009 [14]	16.80 ± 2.57	29.01 ± 2.90		Atenolol	15.90 ± 1.78	18.50 ± 1.78		II grade hypertension
	9.70 ± 1.71	18.94 ± 2.32		Atenolol	9.30 ± 1.76	12.90 ± 1.92		III grade hypertension
Tzemos et al., 2001 [25]			435 ± 27	Atenolol			No difference with baseline	Baseline: 185 ± 39%

A wide variation in the values of FBF were found in the current trials compared with FMD values. The changed FBF value after treatment with nebivolol was 21% in the report of Neuman et al. [13], and this mean changed value was 435% in another study [25]. Pronko et al. [14] detected the FBF value in the patients with II grade and III grade hypertension, respectively. Along with the development of hypertension from II grade to III grade, FBF values ranged from 16.80 ± 2.57 to 9.70 ± 1.71% before treatment and from 29.01 ± 2.90 to 18.94 ± 2.32% after treatment with nebivolol. In the group of control antihypertensive, atenolol, these FMD values ranged from 15.90 ± 1.78 to 9.30 ± 1.76% before treatment and from 18.50 ± 1.78 to 12.90 ± 1.92% after treatment (Table 5).

### Effects of antihypertensives on the endothelial function in the patients with essential hypertension

#### Nebivolol versus other β-blockers

Although lack of detailed data, two trials reported the changed FMD value of the hypertensive patients before and after treatment with nebivolol or carvedilol [20,22]. Both nebivolol and carvedilol had a significantly increased endothelium-dependent vasodilation compared with baseline values, but no statistical difference was found between these treatments [20]. However, in Fedorishina et al.’s report [22], only patients receiving carvedilol showed significantly elevated percentage (from 4.5 to 27.3%) of full recovering of endothelial function (defined as FMD > 10%), suggesting that carvedilol played a better endothelial function-protective effect than nebivolol.

Tzemos et al. [25] reported that nebivolol, rather than atenolol, provided an additional vascular protection in patients with essential hypertension. The percentage change of FBF response to acetylcholine (435 ± 27%) which suggested endothelium-dependent vasodilation was significantly increased in patients received nebivolol compared with baseline (185 ± 39%). However, the present study did not report specific data of percentage change of FBF in patients received atenolol. In another study, effects of nebivolol or atenolol on the endothelial function were compared in the patients with II grade or III grade hypertension, respectively [14]. The results indicated that whatever in the II grade or III grade hypertension, patients received nebivolol treatment had significantly improved endothelial dysfunction, and this effect was not observed after atenolol treatment. Using a method of NO blockade, Neuman et al. [13] reported that the percent change of FBF was 21 and 12% in hypertensive patients with nebivolol and metoprolol, respectively, although no statistical difference was reached (*P*=0.053). The changed FBF response to acetylcholine showed no statistical difference between two treatment groups, either.

**Figure 2:**
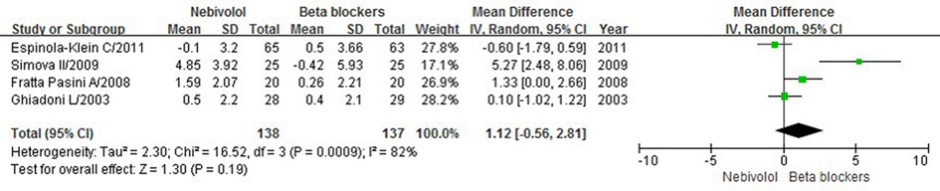
Forest plot of changed FMD values before and after treatment with nebivolol and other β-blockers

Overall, four studies [21,23,24,26] had the complete data for quantitative analysis of the changed FMD value before and after treatment with nebivolol and other β-blockers. All of them evaluated the effect of β-blockers on brachial FMD. A total of 388 patients were included in these trials, in which 138 patients received nebivolol and 137 patients received other β-blockers. Significant heterogeneity was found across these trials (*P=*0.0009, *I*^2^ = 82%), thus, a random-effect model was used to perform the meta-analysis. In these four studies, two used atenolol [23,24] as control drugs, one was bisoprolol [26], and one was metoprolol [21]. The results showed that the changed FMD value of the patients before and after treatment with nebivolol was not statistically different from those in patients treated with other β-blockers [MD = 1.12, 95% confidence interval (CI): −0.56, 2.81, *P*=0.19, Figure 2].

#### Nebivolol versus other classes of antihypertensive drugs

Sendur et al. [11] evaluated some markers of endothelial function after the hypertensive patients (stage I) received nebivolol or olmesartan. The patients had significantly improved endothelial function after antihypertensive treatments, and no statistical difference was found between two antihypertensive drugs. The levels of other markers associated with endothelial function including NO and plasminogen activator inhibitor 1 (PAI-1) was not significantly different either.

Ghiadoni et al. [24] compared the effects of several antihypertensive drugs including nebivolol, nifedipine GITS, amlodipine, telmisartan, and perindopril on FMD of the hypertensive patients before and after 6-month treatments. The results indicated that only perindopril could significantly increase the FMD, suggesting an effect of endothelium-dependent vascular dilation.

## Discussion

Despite the relationship between endothelial dysfunction and essential hypertension has not been fully elucidated, the current evidence indicates that hypertensive patients has decreased endothelial function compared with healthy subjects [24]. Pronko et al. [14] reported that 23.5–27% patients with II grade hypertension had endothelial dysfunction and this incidence was 75% observed in patients with III grade hypertension. It seems that hypertension and endothelial dysfunction are mutually influencing risk factors. Transient hypertension induced by experiment could lead to an acute endothelial dysfunction in normotensive volunteers [27]. In reverse, the alteration of microvascular structures including remodeling and rarefaction are also crucial to the development of hypertension, independent of renal dysfunction [28].

It seems that nebivolol has some special features to alleviate endothelial dysfunction compared with other antihypertensive drugs. The mechanism by which nebivolol improves endothelial function may be partly associated with its β3-adrenoceptor activating effect including vasodilation and antioxidation [16]. In contrast with β1-adrenoceptor, the expression of β3-adrenoceptor is commonly observed in the myocardium and endothelial cells [29]. Activation of β3-adrenoceptor can further up-regulate the expression of endothelial nitric oxide synthase (eNOS) and stimulate the release of NO [30]. Nebivolol plays important protective roles in vasculatures via the regulation of the endothelium-dependent vascular tone through β3-adrenoceptor/eNOS pathway [31]. Thus, nebivolol may provide a better protective effect on endothelial function due to enhanced NO bioavailability compared with other antihypertensive drugs [17,18].

Endothelial function is generally evaluated by its vasodilatory capacity. Two common methods in the evaluation of human vasodilatory function include invasive measurement of the FBF using VOP and non-invasive measurement of FMD. Although FBF measurement is invasive, infusion of vasoactive agents such as acetylcholine during the detection is an advantage of this method. In the current analysis, effects of antihypertensive agents on endothelial function evaluated by both methods are included. Notably, although both FMD and FBF methods are associated with the peripheral vascular endothelium function, FBF mainly reflects the microvascular (resistance artery) endothelium-dependent dilation and FMD assesses the macrovascular (conduit artery) endothelium-dependent dilation [32].

In the present study, basic characteristics of the patients were comparable before treatments between groups. Furthermore, the SBP and DBP were not statistically different after antihypertensive treatments between groups. Quantitative analysis was performed using the complete data from a total of four studies [21,23,24,26], all of them compared the effects of nebivolol with other β-blockers. Unexpectedly, the results of meta-analysis indicated that the changed FMD value of the patients before and after treatment with nebivolol was not statistically different from those in patients treated with other β-blockers including atenolol, bisoprolol and metoprolol, with a *P*-value of 0.19. This result suggests that nebivolol is not a unique endothelial function-protective agent distinguished from other β-blockers.

Simova et al. [26] found a dramatically increased reactivity of the brachial artery (i.e., higher mean difference of FMD values) in hypertensive patients treated with nebivolol rather than bisoprolol, although these agents had similar effect on the blood pressure. Nebivolol has better β-1 selectivity compared with bisoprolol and atenolol [33], and has a unique mechanism to stimulate the l-arginine/NO pathway [34]. In agreement with these findings, Pasini et al. [22] reported that the hypertensive patients showed better endothelial function after treatment with nebivolol compared with atenolol. This effect could be partly explained by weaker antioxidant activity of atenolol compared with nebivolol, which significantly elevate the level of NO via reducing the oxidative inactivation [35]. However, conflicting results are still found in two studies. One of them showed that the changed FMD values were not statistically different between nebivolol and atenolol treatment, and both drugs could not modify FMD in the brachial artery of the hypertensive patients [24]. In the other study, Espinola-Klein et al. [21] reported that after a treatment period of 48 weeks, no significant change of FMD values was found in nebivolol group compared with baseline, and no statistical difference of changed FMD values noted between nebivolol group and metoprolol group.

Be short of detailed original data about the values of FMD or FBF before and after treatment, several studies could not be quantitatively analyzed. In two studies comparing the endothelial function-protective effects between nebivolol and atenolol using FBF method, they all tell the same story. Tzemos et al. [25] reported that nebivolol could provide an additional vascular protection in patients with essential hypertension compared with atenolol. The other report indicated that patients with II grade or III grade hypertension received nebivolol treatment showed significantly improved endothelial dysfunction, and this effect was not observed after atenolol treatment [14]. Using FBF method, similar result is also found between nebivolol and metoprolol treatment compared with FMD detection. Neuman et al. [13] reported that although a changed FBF value was 21% in hypertensive patients with nebivolol and this value was 12% after metoprolol treatment, no statistical difference was noted (*P*=0.053). The result of qualitative synthesis also suggested that carvedilol and perindopril could significantly increase the FMD compared with nebivolol, resplectively [22,24]. However, the result is still conflicting in Zepeda et al.’s report, in which no statistical difference was found between nebivolol and carvedilol treatments [20].

Impaired vasodilation response to specific stimulus such as acetylcholine is a remarkable feature of endothelial dysfunction. Taddei et al. [36] identified a genetic determination of endothelial dysfunction in hypertensive patients. Interestingly, compared with the normotensive offspring of normotensive volunteers, the normotensive offspring of the hypertensive patients showed an impaired response to acetylcholine, suggesting an early-stage endothelial dysfunction before the development of hypertension. Hypertensive patients with improved endothelial function showed better outcome after antihypertensive treatments compared with those had endothelial dysfunction [8]. A Framingham study including 2883 participants indicated that SBP was inversely correlated with FMD [37]. Rossi et al. [38] reported that postmenopausal normotensive women with low FMD had significantly higher risk of development of hypertension compared with those with high FMD. Currently, a new emerging concept indicates that the long-term regulation and maintenance of systemic blood pressure is critically associated with the peripheral microvascular tone [39,40].

In the current study, we have summarized several antihypertensive drugs including nebivolol, carvedilol, metoprolol, atenolol, olmesartan, and perindopril possessing the power to improve the endothelial function, although the present data could not finally conclude that which one is better. The characteristic of nebivolol may provide a great potential application in protecting endothelial function of the hypertensive patients, and it may translate to a long-term prognostic benefit in future. However, the current topic limits the number of included trial subjects, in addition to the relative wide variations of brachial FMD or FBF values among studies, leading to a meta-analysis result of non-statistical difference. Endothelial dysfunction is associated with the development of cardiovascular complications of the hypertension, and rectifying endothelial dysfunction will improve the prognosis of the patients with essential hypertension [14]. Currently, endothelial function still cannot be used to guide hypertension treatment instead of blood pressure. However, the clinical significance of protection of endothelial function for hypertension treatment will arouse the attention of the physicians, and we believe that more accurate conclusions could be drawn in future on the basis of more detailed data and standardized measuring methods.

In conclusion, nebivolol is not a unique endothelial function-protective agent distinguished from other β-blockers or other classes of antihypertensive drugs, a large-scale clinical trials in future are required to draw an accurate conclusion. Improvement of endothelial function is a key point in the prevention and therapy of essential hypertension. Antihypertensive drugs with reversal of endothelial dysfunction should be recommended in the treatment of essential hypertension.

## Abbreviations

CI: confidence interval
DBP: diastolic blood pressure
eNOS: endothelial nitric oxide synthase
FBF: forearm blood flow
FMD: flow-mediated vasodilatation
NO: nitric oxide
SBP: systolic blood pressure
VOP: venous occlusion plethysmography
